# *Helicobacter canis* bacteraemia and cellulitis in a patient with end-stage renal disease

**DOI:** 10.1099/jmmcr.0.005126

**Published:** 2017-11-08

**Authors:** Salika M. Shakir, Margaret V. Powers-Fletcher, E. Susan Slechta, Mark A. Fisher

**Affiliations:** ^1^​ Department of Pathology, University of Utah School of Medicine/ARUP Laboratories, Salt Lake City, UT, USA; ^2^​ Department of Pathology, University of Cincinnati Medical Center, Cincinnati, OH, USA; ^3^​ ARUP Institute for Clinical and Experimental Pathology, Salt Lake City, UT, USA

**Keywords:** *Helicobacter canis*, zoonosis, bacteraemia, cellulitis, 16S rRNA sequencing, intervening sequence, underlying disease

## Abstract

**Introduction.** Invasive infections by *Helicobacter canis* are uncommon and occur primarily in immunocompromised patients. Here, we describe a case of *H. canis* bacteraemia and cellulitis in a patient with end-stage renal disease (ESRD).

**Case presentation.** A 49-year-old male with ESRD on haemodialysis presented to an emergency department with cellulitis overlying his left upper extremity arteriovenous fistula for 3 days without constitutional symptoms. Mild leucocytosis and thrombocytopenia was noted on initial laboratory work up. The patient received a dose of vancomycin initially, and then transitioned to oral doxycycline prior to discharge 3 days later. Blood cultures drawn on admission were positive with curved Gram-negative rods at day 5. Routine sub-cultures initially failed to isolate the organism; however, small, tan colonies were observed on sheep blood agar incubated under microaerobic conditions. *H. canis* was identified by 16S rRNA sequencing. Antimicrobial-susceptibility testing was not performed due to poor growth and lack of interpretive guidelines. The patient was ultimately treated successfully with amoxicillin/clavulanic acid.

**Conclusion.** This case illustrates the importance of recognizing *H. canis* infections in immunocompromised patients, especially in those with recent pet exposure. In addition, this case highlights the need for improved laboratory diagnostics for *H. canis* as isolation and identification of this fastidious organism is challenging.

## Abbreviations

A/C, amoxicillin/clavulanic acid; ESRD, end-stage renal disease; IVS, intervening sequence; MALDI-TOF, matrix-assisted laser desorption ionization–time of flight.

## Introduction

*Helicobacter canis*, a member of the enterohepatic helicobacters, inhabits the lower intestinal and hepatobiliary tract of dogs and cats. These bacteria were first described in 1993 by Stanley *et al*. as *Campylobacter*-like organisms (CLOs) isolated from the faeces of both diarrhoeic and healthy dogs and cats, and as the dominant *Helicobacter* species in canine saliva [1, 2]. An extensive literature search yielded eight human cases of *H. canis* to date in patients with underlying conditions. It was first described from a boy with gastroenteritis and subsequently from blood cultures in a patient with X-linked hypogammaglobulinaemia [3, 4]. It has been reported in a renal transplant patient and in a patient with gastric lymphoma [5, 6]. Five of the eight cases/reports described bacteraemia in immunocompromised patients following close contact with dogs or cats [4–8].

Isolation and recognition of *H. canis* is challenging under routine laboratory conditions. Due to its fastidious nature and relatively inactive biochemical profile, special culture conditions and molecular methods are important tools in the diagnosis of *H. canis* infections. Here, we present a case of bacteraemia and cellulitis caused by *H. canis* in a patient with end-stage renal disease (ESRD) who was in close contact with his pet dog.

## Case report

A 49-year-old male with a history of ESRD on haemodialysis for 8 years, hypertension and chronic pancreatitis, presented to an emergency department with a 2 day history of worsening pain, swelling and erythema of his left upper extremity overlying an arteriovenous fistula. The patient lacked constitutional symptoms and was haemodynamically stable, but had mild leucocytosis (white blood cells 1.32×10^4^ cells µl^−1^), thrombocytopenia (1.54×10^5^ cells µl^−1^) and elevated C-reactive protein (9.4 mg dl^−1^). Vascular ultrasound demonstrated no venous or arterial thrombotic complications. However, due to concern for staphylococcus cellulitis the patient was given one dose of intravenous vancomycin (1.5 g) in the emergency department. Erythema regressed by day 3, and the patient was discharged on oral doxycycline (100 mg twice daily for 1 week).

**Fig. 1.**
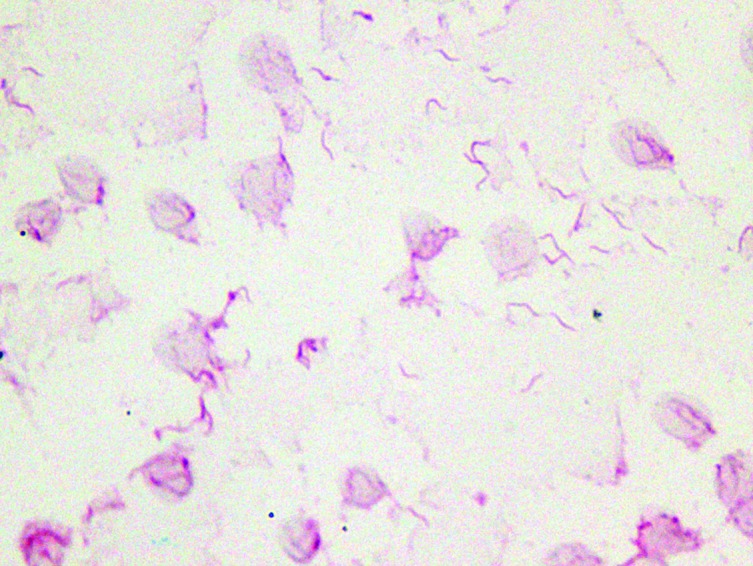
A Gram stain of the positive blood culture showing the curved Gram-negative rods of *H. canis* (original magnification ×1000; oil immersion).

On day 5, both sets of aerobic blood culture bottles drawn on admission were reported positive for small, curved Gram-negative rods (Fig. 1). Bottles were sub-cultured onto 5 % sheep blood, chocolate and MacConkey agar plates, which failed to grow in 5 % CO_2_ by 48 h. Additional sub-cultures were plated on sheep blood agar and incubated microaerobically using the AnaeroPack – MicroAero system (Mitsubishi) at 35 and 42 °C, and small, tan colonies grew after 72 h. Matrix-assisted laser desorption ionization–time of flight (MALDI-TOF) MS failed to identify the organism. It was identified as *H. canis* by partial 16S rRNA gene sequencing. In brief, PCR amplification and sequencing of the partial 16S rRNA gene was performed with primers 5F-t and 534R-t [9]. Sequences were assembled and analysed with Ripseq (Pathogenomix), which showed a poor match to the *H. canis* type strain. The sequence was then compared with all 16S rRNA sequences available in GenBank by using the blastn 2.6.0 program and demonstrated 99.7 % identity over 673 nt to a *H. canis* isolated from a dog liver with multifocal necrotizing hepatitis (GenBank accession no. U65102). Antimicrobial-susceptibility testing was not performed due to poor growth; however, therapy was changed from doxycycline to 8 weeks of oral amoxicillin/clavulanic acid (A/C) (500 mg, daily), which resulted in a bacteriological and clinical cure.

## Discussion

As a dominant helicobacter in the alimentary tract of the companion animals, dogs and cats, it is not surprising that several cases of *H. canis* have been described in the literature. A PubMed/Medline search (keywords: *Helicobacter canis*, clinical case, bacteremia) revealed eight published cases of human infections with *H. canis*. Although the first described case was of gastroenteritis [3], the majority of subsequent cases were of bacteraemia in immunocompromised patients. Similar to our patient, bacteraemia in five of the reported cases followed contact with dogs or cats, and two were associated with cellulitis, as seen in our patient [4, 7]. These similarities highlight the likely zoonotic potential of *H. canis* and suggest that cellulitis in an immunocompromised patient may be an indicator of *H. canis* infection.

**Fig. 2.**
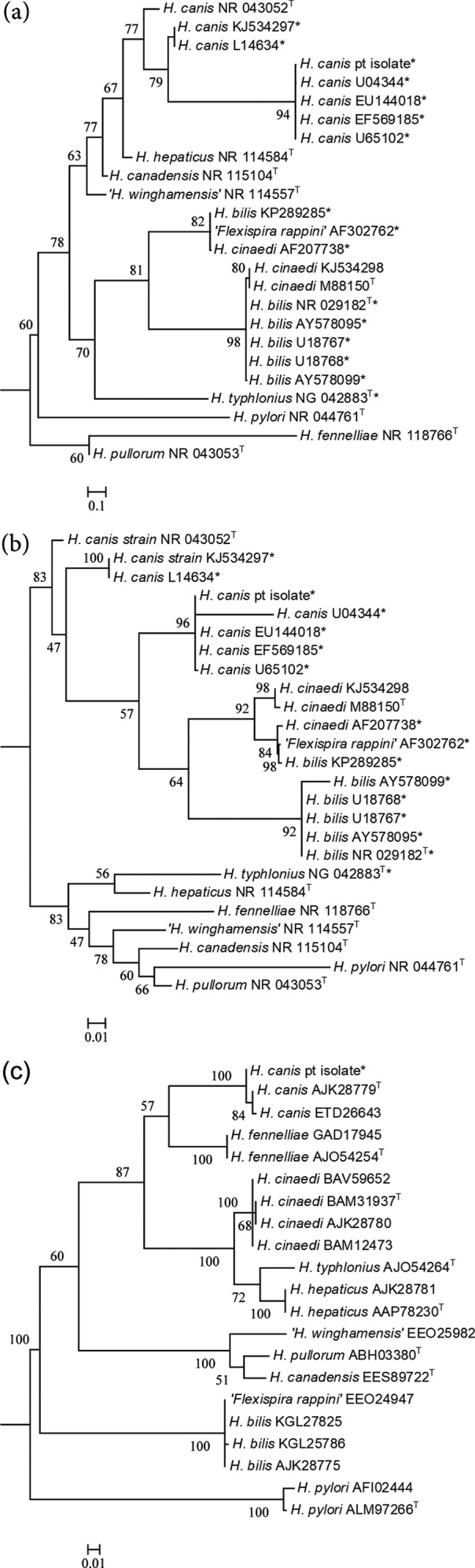
Maximum-likelihood phylogenetic trees showing the relationship of the patient’s isolate to other *Helicobacter* species. (a) Partial 16S tree based on the assembly of approximately 676 bp of the 5′ 16S rRNA gene. (b) Phylogenetic tree based on the alignment of near-full length 16S rRNA gene sequences (approximately 1678 bp). (c) Phylogenetic tree of partial GyrA protein sequences (approximately 352 aa/1060 bp). Alignments and the tree were generated with ClustalW and PhyML, and bootstrap analysis was performed with 100 resamplings [11]. Type strains are denoted with a T. Strains with an IVS are represented with an asterisk.

Because of its fastidious nature, isolation of *H. canis* may be difficult without special culture conditions. Although *H. canis* can grow in routine aerobic blood culture bottles in 3–5 days, microaerobic conditions (35 or 42 °C) are generally required for its isolation from stool and positive blood cultures. Once isolated, identification is challenging due to the weak reactivity of the bacteria in biochemical panels. Because of this, molecular methods are important tools in the diagnosis of *H. canis* infections. 16S rRNA gene sequence analysis has been more reliable than MALDI-TOF for definitive identification, and can be used on positive blood cultures for isolates that fail to grow in sub-culture. Interestingly, a 235 bp intervening sequence (IVS) was identified in the 16S rRNA gene of our patient’s isolate. Some *H. canis* isolates carry this sequence of unknown function, but many, including the type strain, lack the IVS [10]. This isolate was 99.7 % identical over 99 % of the partial 16S sequence (including the IVS) to a canine necrotizing hepatitis *H. canis* isolate (GenBank accession no. U65102). The *H. canis* type strain (ATCC 51401) showed 99–100 % identity to the regions flanking the IVS, but due to the lack of an IVS, showed only a 64 % identity to our isolate. Phylogenetic analysis of the partial 16S rRNA gene from our patient’s isolate using clustalw and PhyML (bootstrapped with 100 resamplings) on default settings at Phylogeny.fr [11] showed that it clusters most closely with *H. canis* isolates other than the type strain, namely those isolates containing IVSs (Fig. 2a). These data illustrate that the IVS found in some *H. canis* strains may confound 16S sequence-based identification when only type strain databases or databases of limited diversity are used. The identification of our isolate as *H. canis* was confirmed with nearly complete 16S rRNA gene (1678 bp using 5F and 1492R primers [9]) (Fig. 2b), and partial *gyrA* gene (1060 bp using primers for PCR numbers 2–3 from Menard *et al*. [12]) sequence analysis as described for the partial 16S analysis and using the ‘Quick Bioinformatic Phylogeny of Prokaryotes’ software (leBIBI, v1.1) under stringent conditions [13]. Menard *et al.* noted the relationships among *Helicobacter* species were well defined with the GyrA protein sequence, and this approach, using ClustalW alignment and PhyML on default settings at Phylogeny.fr [11], also supported the identification of our isolate as *H. canis* (Fig. 2c; 99 % identical at the protein level to *H. canis* strain NCTC 12740, GenBank accession no. ETD26643). The near full length 16S rRNA gene and partial *gyrA* sequences have been deposited in GenBank (accession numbers: MF542253 and MF953398, respectively).

Susceptibility testing for *H. canis* is not well defined and no interpretative guidelines are available. Outcomes of *H. canis* infections using different antimicrobial therapies vary among cases in the literature. In this case, 7 days of doxycycline followed by 8 weeks of oral A/C successfully resolved the infection. This is in contrast with a prior report that suggested A/C was suboptimal for the treatment of *H. canis* bacteraemia and cellulitis [7]. In that case, an immunocompetent patient had a recurrence of plaques 2 days after completion of a 10 day course of A/C, and intravenous ceftriaxone (2 g day^−1^, 2 weeks) was required to resolve the infection. Two additional case reports described positive outcomes with oral doxycycline (combined with metronidazole for 5 months or 6 weeks doxycycline plus 2 weeks intravenous ceftriaxone), and a third had success with 3 days of intravenous cefuroxime followed by oral ciprofloxacin for 10 days [4, 6, 14].

In conclusion, *H. canis* infections are difficult to diagnose and are thus likely under-recognized. This case illustrates that clinicians should recognize the role of this organism in cases of bacteraemia and cellulitis in patients with underlying comorbidities, especially those with recent pet exposure, who are not improving with standard treatment for cellulitis. Consultation with an experienced clinical microbiologist is important to guide the use of optimal culture conditions and appropriate molecular identification methods when Gram stain results compatible with *H. canis* are encountered.
